# Heralding Extramedullary Blast Crisis: Horner's Syndrome with Brachial Plexopathy in a Patient with Chronic Myelogenous Leukemia

**DOI:** 10.1155/2016/3015947

**Published:** 2016-12-21

**Authors:** Sajish Jacob, Sadanand I. Patil

**Affiliations:** ^1^Memphis Neurology, 1407 Union Avenue, No. 1400, Memphis, TN 38014, USA; ^2^Baptist Cancer Center, 6029 Walnut Grove Rd, No. 300, Memphis, TN 38120, USA

## Abstract

Chronic myelogenous leukemia (CML) blast crisis is an ominous clinical event that is challenging to treat. This can develop at extramedullary sites rarely and is defined as the infiltration of blasts outside the bone marrow irrespective of proliferation of blasts within the bone marrow. We aim to report an unusual clinical presentation characterized by Horner's syndrome, ipsilateral arm weakness, and cervical lymphadenopathy as the first signs of extramedullary blast crisis in a CML patient. To the best of our knowledge, the extramedullary locations involving the brachial plexus along with cervicothoracic paraspinal chloroma have not been previously reported in the literature.

## 1. Introduction

Chronic myelogenous leukemia (CML) is classified as a myeloproliferative neoplasm which usually progresses from a relatively indolent disease to a more aggressive disorder, during which time disease control is harder to achieve [1]. In virtually all patients, the disease culminates into an acute leukemic phase, especially if untreated, termed “blast crisis.” Blast crisis of CML is an ominous clinical event that can further be divided based on the site of origin: medullary or extramedullary [2]. Extramedullary blast crisis is extremely rare and is defined as the development of extramedullary blasts infiltration irrespective of the proliferation of blasts in the bone marrow [3]. We report an unusual clinical presentation heralding blast crisis transformation in a CML patient, characterized by cervical lymphadenopathy, cervicothoracic paraspinal chloroma with leukemic infiltration of the brachial plexus causing left Horner's syndrome, and ipsilateral symptomatic arm involvement.

## 2. Case Presentation

A 36-year-old African American male diagnosed with Philadelphia chromosome positive CML two years ago presented to the emergency room for the second time with a history of a one-month progressively worsening weakness involving his left upper extremity, along with numbness and severe pain. His symptoms during the previous hospital evaluation were attributed to musculoskeletal causes after stroke was ruled out. Although he was unsure, he was reported by family members to have developed some facial asymmetry during this time. He had been noncompliant with his treatment with dasatinib, being irregular and casual in his approach to prescribed medication intake, and his primary oncologist was concerned with CML progression at an accelerated phase after having undergone a bone marrow biopsy. He was meanwhile awaiting the results of a recent cervical lymph node biopsy.

**Figure 1:**
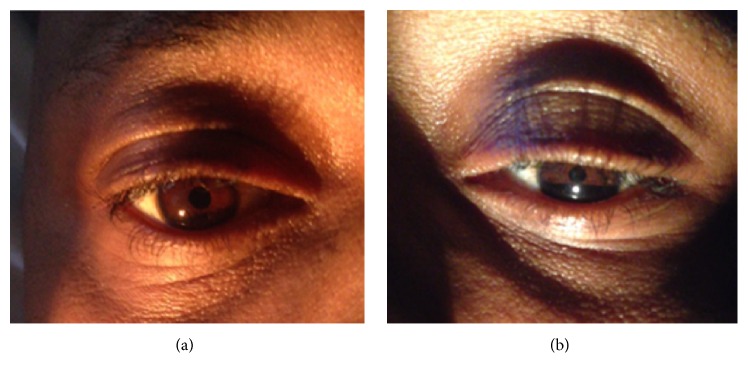
Patient with left Horner's syndrome: normal right eye (a) and external examination in comparison to left eye (b) with ptosis, miosis, and enophthalmos.

On neurological examination the patient was noticed to have left-sided ptosis, miosis, enophthalmos (Figure 1), and reported facial anhidrosis on the left side, alongside ipsilateral weakness predominantly involving his intrinsic hand muscles, long finger flexors, and numbness involving the medial border of his arm, hand, and little finger (see Video 1 in Supplementary Material available online at http://dx.doi.org/10.1155/2016/3015947). The rest of his motor power was 5/5 throughout, with restriction of an accurate evaluation of his left upper extremity, due to exquisite pain-limited range-of-motion of the whole limb. Deep tendon reflexes were 2+ throughout. Plantar reflexes were down going bilaterally and no sensory level was appreciated. His findings were concerning for left Horner's syndrome and lower trunk brachial plexopathy, while the rest of his general physical examination was significant for scattered, nontender cervical lymphadenopathy.

**Figure 2:**
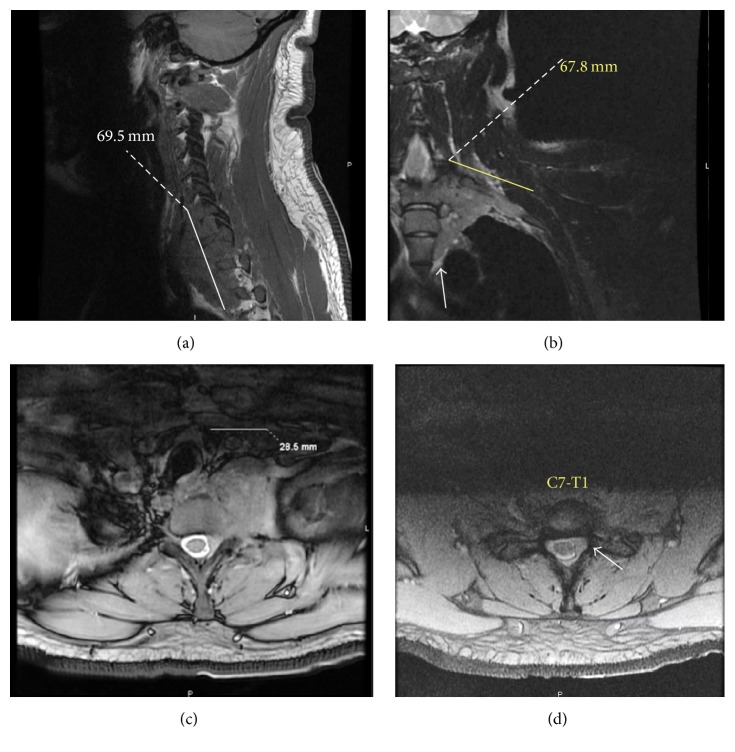
Magnetic Resonance Imaging (MRI) of the cervical spine and brachial plexus at cervical-thoracic levels: T2-weighted images with left paraspinal soft tissue mass and brachial plexus infiltration in sagittal (a), coronal (b), and axial (c) views and intraspinal extension (d).

**Figure 3:**
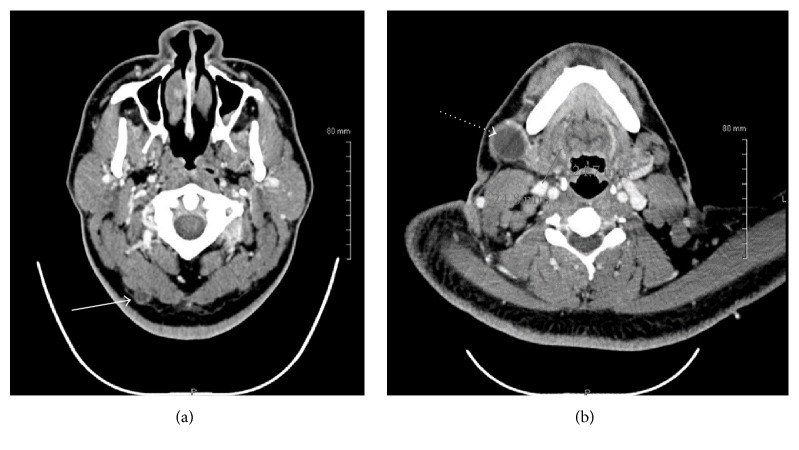
Computed Tomography (CT) scan of soft tissue neck demonstrating right occipital (solid arrow) and right jugulomandibular (dotted arrow) cervical lymphadenopathies.

**Figure 4:**
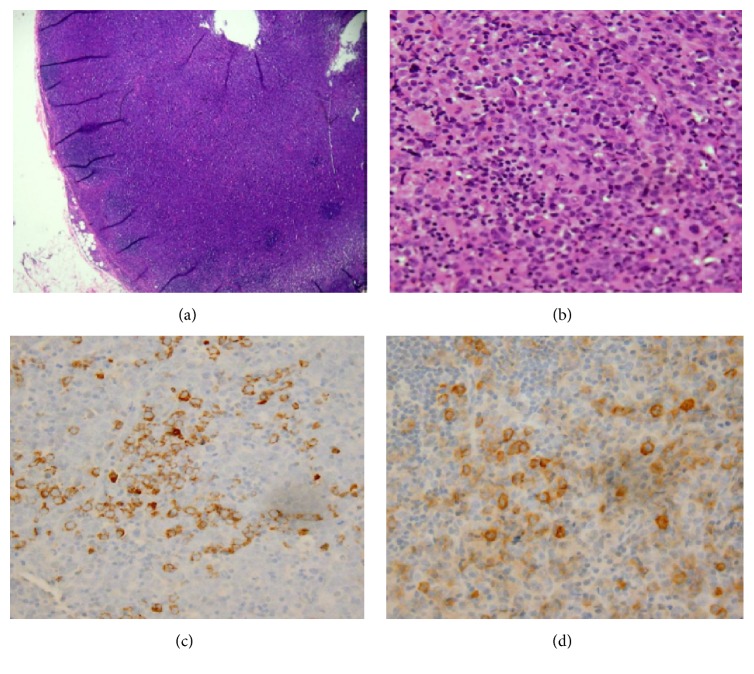
Histopathological analysis. (a) Low magnification view with effacement of lymph node architecture. (b) High magnification view with numerous immature cells (blasts). (c) A proportion of the immature cells are positive for myeloperoxidase by immunohistochemistry. (d) A proportion of the immature cells are positive for CD117 by immunohistochemistry.

His blood count revealed marked leukocytosis with a total leukocyte count of 102 × 10^9^/L, predominated by myeloid precursors, 4% promyelocytes, 3% myelocytes, and 12% metamyelocytes. An electrodiagnostic study was deferred due to lack of patient cooperation from the pain. An MRI of the cervical spine and brachial plexus with and without contrast was performed. This revealed the presence of a bulky left paraspinal soft tissue mass (Figure 2), extending from the C6 to T3 level, measuring 3.9 × 6.8 × 7.8 cm, anteroposterior, transverse, and cephalocaudad dimensions, respectively. This also involved the left C6 to T1 nerve roots and extended into the supraclavicular and retroclavicular segment of the left brachial plexus over approximately 6.8 cm in length, most consistent with leukemic infiltration. Extension into the spinal canal on the left was noted from C6/7 to the bottom of the T1 level without cord compression, along with abnormal bone marrow signal intensity involving the posteromedial first rib and left anterior scalene muscle, all again consistent with leukemic infiltration. Extensive lymphadenopathy was noted on imaging studies (Figure 3), particularly involving the submandibular, left, and right internal jugular lymph nodes. The excisional biopsy of right cervical lymph node, meanwhile, confirmed myeloid sarcoma or chloroma with immunophenotyping revealing 32% blasts displaying myeloid-associated antigen expression and aberrant expression of CD5, CD7, and CD 117 (Figure 4). In view of the collateral histological findings, his neurological presentation was attributed to a paraspinal chloroma with regional leukemic infiltration of the left lower brachial plexus, and a final diagnosis of CML with extramedullary myeloid blast crisis was made. He was started on aggressive induction chemotherapy with a 7 + 3 regimen (7 days of cytarabine at 100 mg/m^2^ plus 3 days of idarubicin at 12 mg/m^2^) in addition to resuming dasatinib. He was also initiated on radiotherapy of 10 fractionations to a total of 3000 centigray (cGy) for the paraspinal chloroma. Bone marrow biopsy on Day 14 was hypocellular with no blasts identified or evidence of residual leukemia. Repeat Computed Tomography of soft tissue neck showed interval decrease in size of his cervical lymphadenopathies and paraspinal soft tissue mass. His postchemotherapy, inhospital course was complicated with neutropenic fever, clostridium difficile diarrhea, and multifocal pneumonia with pleural effusion from which he gradually recovered. At the time of hospital discharge, his neurological deficits continued to persist. Allogeneic bone marrow transplantation and prophylactic intrathecal liposomal cytarabine (given his high risk for CNS involvement) were discussed as part of the postremission treatment plan.

## 3. Discussion

Horner's syndrome (also called oculosympathetic paresis) typically consists of miosis, ptosis, and hemifacial anhidrosis and is a well-described neurological syndrome that is useful clinically for neurological localization. Any lesion along the sympathetic tract that supplies the eye, head, and neck can result in Horner's syndrome [4]. The ptosis observed occurs due to involvement of the Mueller's muscle in the upper eyelid. Also the lower lid is slightly elevated (upside-down ptosis) by a few millimeters creating an appearance of enophthalmos or sunken eyes. Unopposed parasympathetic activity on the pupillary constrictors produces miosis in the affected eye. Asymmetry in the pupils or anisocoria is more apparent in dim light. Fibers controlling facial sweating travel along the external carotid artery and hence lesions distal to the carotid bifurcation spare facial sweating, while those proximal to the carotid bifurcation can cause anhidrosis. Clinical findings other than the classic triad can also be observed including pupillary dilation lag, loss of ciliospinal reflex, and bloodshot conjunctiva from vasodilation [5]. For localization purposes it can be divided into first-order, second-order, and third-order syndromes. A first-order or central Horner's syndrome can be produced by lesions in the brainstem or cervicothoracic spinal cord affecting sympathetic tracts. These central nervous system lesions are usually associated with collateral long tract neurologic signs, visual field deficits, double vision, or myelopathic features. Second-order or preganglionic (proximal to the superior cervical ganglion) Horner's syndrome is usually seen with neck trauma, thoracic outlet lesions, thyroid malignancies, or a Pancoast tumor. It typically involves the lower brachial plexus and ipsilateral arm pain often accompanies the Horner's syndrome in such cases [6]. Third-order or postganglionic (at or distal to the superior cervical ganglion) Horner's syndrome often indicates lesions of the internal carotid artery, typically seen with aneurysm or carotid dissection and generally spares facial sweating. Other causes include cavernous sinus thrombosis, middle ear infection, neck masses, and cluster or migraine headaches.

In our patient, the presentation of Horner's syndrome with associated left arm pain, weakness, and sensory deficit was localized to a second-order syndrome caused from extramedullary chloroma and leukemic infiltration of the brachial plexus.

As per definition, CML blast phase can be diagnosed if an extramedullary blast proliferation is identified (i.e., myeloid sarcoma or chloroma). It can also be diagnosed if the blast count is at least 20% of the white blood cells in the peripheral blood or in the nucleated cells in the bone marrow [3, 7]. CML in chronic phase can progress to blast crisis at a rate of approximately 1% per year. The two major forms are divided based on the type of cell into myeloid or lymphoid. The most common sites of extramedullary involvement in CML are lymph nodes and bones followed by the central nervous system [2, 8, 9]. Blast crisis remains a challenging problem in the management of CML. As advances are made in the treatment of the chronic phase of CML, fewer patients are progressing to the accelerated phase and blast crisis. Once blast crisis in CML has been diagnosed, an attempt is made to return the patient to a chronic phase. All patients should undergo a Philadelphia chromosome or BCR-ABL kinase mutational profiling and be evaluated for allogeneic hematopoietic cell transplantation (HCT), as this offers the best chances for long-term survival. Initial treatment with tyrosine kinase inhibitor (based on mutation analysis) is advocated and continued in patients who show a treatment response [10–12]. If an initial tyrosine kinase inhibitor (TKI) is not tolerated or if there is a lack of response to treatment, an alternate TKI or TKI plus acute leukemia-type induction chemotherapy should be followed through [13].

## 4. Conclusion

The treatment for Horner's syndrome depends upon the cause and is generally aimed at addressing the disease that is involved in the syndrome. In our patient, the presentation of Horner's syndrome with associated arm pain, weakness, and sensory deficit localized the level of his lesion to a second-order syndrome. Further targeted investigations led to the diagnosis of CML in extramedullary blast crisis. It is important for physicians to recognize Horner's syndrome, understand its anatomical pathways and associated collateral clinical features for accurate localization, and avoid delay in diagnosis, which can have important neurological prognostic consequences. Moreover, it is important to maintain a high index of suspicion in patients with a history of CML, and a Horner's syndrome could indicate the presence of an extramedullary blast crisis.

## Supplementary Material

Video in the Supplementary Material briefly includes relevant eye findings and muscle strength of the left upper extremity of the patient.

## References

[1] Alimena G., Breccia M., Latagliata R. (2006). Sudden blast crisis in patients with Philadelphia chromosome-positive chronic myeloid leukemia who achieved complete cytogenetic remission after imatinib therapy. *Cancer*.

[2] Sahu K. K., Malhotra P., Uthamalingam P. (2016). Chronic myeloid leukemia with extramedullary blast crisis: two unusual sites with review of literature. *Indian Journal of Hematology and Blood Transfusion*.

[3] Vardiman J. W., Harris N. L., Brunning R. D. (2002). The World Health Organization (WHO) classification of the myeloid neoplasms. *Blood*.

[4] Edwards A., Andrews R. (2001). A case of Brown-Sequard syndrome with associated Horner's syndrome after blunt injury to the cervical spine. *Emergency Medicine Journal*.

[5] Pilley S. F. J., Thompson H. S. (1975). Pupillary ‘dilatation lag’ in Horner's syndrome. *British Journal of Ophthalmology*.

[6] Maloney W. F., Younge B. R., Moyer N. J. (1980). Evaluation of the causes and accuracy of pharmacologic localization in Horner's syndrome. *American Journal of Ophthalmology*.

[7] Levy R. A., Mardones M. A., Burch M. M., Krause J. R. (2014). Myeloid sarcoma as the presenting symptom of chronic myelogenous leukemia blast crisis. *Proceedings (Baylor University. Medical Center)*.

[8] Specchia G., Palumbo G., Pastore D., Mininni D., Mestice A., Liso V. (1996). Extramedullary blast crisis in chronic myeloid leukemia. *Leukemia Research*.

[9] Inverardi D., Lazzarino M., Morra E. (1990). Extramedullary disease in PH'-positive chronic myelogenous leukemia: frequency, clinical features and prognostic significance. *Haematologica*.

[10] Hehlmann R. (2012). How I treat CML blast crisis. *Blood*.

[11] Hehlmann R., Saussele S. (2008). Treatment of chronic myeloid leukemia in blast crisis. *Haematologica*.

[12] Silver R. T. (2009). The blast phase of chronic myeloid leukaemia. *Best Practice & Research Clinical Haematology*.

[13] Axdorph U., Stenke L., Grimfors G. (2002). Intensive chemotherapy in patients with chronic myelogenous leukaemia (CML) in accelerated or blastic phase—a report from the Swedish CML Group. *British Journal of Haematology*.

